# Habitat productivity and pyrethroid susceptibility status of *Aedes aegypti* mosquitoes in Dar es Salaam, Tanzania

**DOI:** 10.1186/s40249-017-0316-0

**Published:** 2017-06-09

**Authors:** Leah Mathias, Vito Baraka, Anitha Philbert, Ester Innocent, Filbert Francis, Gamba Nkwengulila, Eliningaya J. Kweka

**Affiliations:** 1 Department of Zoology and Wildlife Conservation, College of Natural and Applied Sciences, University of Dar es Salaam, P.O. Box 35165, Dar es Salaam, Tanzania; 2 National Institute for Medical Research, Tanga Medical Research Centre, P.O. Box 5004, Tanga Urban, Tanga, Tanzania; 3 Global Health Institute, University of Antwerp, Gouverneur Kinsbergencentrum, Doornstraat 331, B-2610 Wilrijk, Belgium; 4 Mkwawa University College of Education, Private Bag, Iringa, Tanzania; 5 Institute of Traditional Medicine, Muhimbili University of Health and Allied Sciences, P.O. Box 65001, Dar es Salaam, Tanzania; 6 Department of Medical Parasitology and Entomology, Catholic University of Health and Allied Sciences, P.O. Box 1464, Mwanza, Tanzania; 7 Division of Livestock and Human Diseases Vector Control, Tropical Pesticides Research Institute, Ngaramtoni, Off Nairobi Road, P.O. Box 3024, Arusha, Tanzania

**Keywords:** Culicidae, *Aedes aegypti*, Abundance, Productivity, Pyrethroid, Knockdown effect, Dar es Salaam, Tanzania

## Abstract

**Background:**

*Aedes aegypti* (Diptera: Culicidae) is the main vector of the dengue virus globally. Dengue vector control is mainly based on reducing the vector population through interventions, which target potential breeding sites. However, in Tanzania, little is known about this vector’s habitat productivity and insecticide susceptibility status to support evidence-based implementation of control measures. The present study aimed at assessing the productivity and susceptibility status of *A. aegypti* mosquitoes to pyrethroid-based insecticides in Dar es Salaam, Tanzania.

**Methods:**

An entomological assessment was conducted between January and July 2015 in six randomly selected wards in Dar es Salaam, Tanzania. Habitat productivity was determined by the number of female adult *A. aegypti* mosquitoes emerged per square metre. The susceptibility status of adult *A. aegypti* females after exposure to 0.05% deltamethrin, 0.75% permethrin and 0.05% lambda-cyhalothrin was evaluated using the standard WHO protocols. Mortality rates were recorded after 24 h exposure and the knockdown effect was recorded at the time points of 10, 15, 20, 30, 40, 50 and 60 min to calculate the median knockdown times (KDT_50_ and KDT_95_).

**Results:**

The results suggest that disposed tyres had the highest productivity, while water storage tanks had the lowest productivity among the breeding habitats Of *A. aegypti* mosquitoes. All sites demonstrated reduced susceptibility to deltamethrin (0.05%) within 24 h post exposure, with mortalities ranging from 86.3 ± 1.9 (mean ± SD) to 96.8 ± 0.9 (mean ± SD). The lowest and highest susceptibilities were recorded in Mikocheni and Sinza wards, respectively. Similarly, all sites demonstrated reduced susceptibility permethrin (0.75%) ranging from 83.1 ± 2.1% (mean ± SD) to 96.2 ± 0.9% (mean ± SD), in Kipawa and Sinza, respectively. Relatively low mortality rates were observed in relation to lambda-cyhalothrin (0.05%) at all sites, ranging from 83.1 ± 0.7 (mean ± SD) to 86.3 ± 1.4 (mean ± SD). The median KDT_50_ for deltamethrin, permethrin and lambda-cyhalothrin were 24.9–30.3 min, 24.3–34.4 min and 26.7–32.8 min, respectively. The KDT_95_ were 55.2–90.9 min for deltamethrin, 54.3–94.6 min for permethrin and 64.5–69.2 min for lambda-cyhalothrin.

**Conclusions:**

The productive habitats for *A. aegypti* mosquitoes found in Dar es Salaam were water storage containers, discarded tins and tyres. There was a reduced susceptibility of *A. aegypti* to and emergence of resistance against pyrethroid-based insecticides. The documented differences in the resistance profiles of *A. aegypti* mosquitoes warrants regular monitoring the pattern concerning resistance against pyrethroid-based insecticides and define dengue vector control strategies.

**Electronic supplementary material:**

The online version of this article (doi:10.1186/s40249-017-0316-0) contains supplementary material, which is available to authorized users.

## Multilingual abstracts

Please see Additional file 1 for translations of the abstract into the five official working languages of the United Nations.

## Background

Dengue fever is a widespread vector-borne viral disease and is the tropical disease with the fastest global spread recently. *Aedes* spp*.* and dengue infections are highly prevalent in Latin America, Southern Asia and the Caribbean, and also prevalent in Sub-Saharan Africa with about 400 million dengue cases occurring annually (see Table 1) [1, 2]. The disease is the most common arbovirus infection globally, with infections and transmission occurring in at least 128 countries, putting almost four billion people at risk worldwide [2]. The number of dengue cases reported per year is 50 to 100 million cases in over 100 endemic countries [3]. Asia bears the biggest burden of dengue in the world, accounting for 70% of all cases (67 million infections), and is characterised by large belts of highly populated regions with very high conducive environments for dengue transmission [4]. By 2010, Africa had a total of 15 million cases of dengue [4]. The current global burden of the disease estimate death due to dengue to be more than 14 000 people in 2010 [5].

**Table 1:** The Geographic distribution of spatially unique occurrence records for the Americas, Europe/Africa, and Asia/Oceania

	Country	Occurrences
*Ae.aegypti*		
Americas	Brazil	5 044
	USA	436
	Mexico	411
	Cuba	177
	Argentina	170
	Trinidad and Tobago	152
	Venezuela	130
	Colombia	128
	Puerto Rico	120
	Peru	89
*Ae. albopictus*		
Americas	Brazil	3 441
	USA	1 594
	Mexico	50
	Cayman Islands	15
	Haiti	13
	Guatemala	12
	Venezuela	7
	Colombia	3
	Cuba	3
	Puerto Rico	3
Europe/Africa	Senegal	112
	Cameroon	55
	Kenya	52
	United Republic of Tanzania	44
	Cote d’Ivoire	40
	Nigeria	35
	Madagascar	28
	Gabon	27
	Mayotte	20
	Sierra Leone	20
Europe/Africa	Italy	203
	Madagascar	58
	Cameroon	42
	France	37
	Gabon	27
	Albania	22
	Mayotte	21
	Greece	18
	Israel	17
	Lebanon	15
Asia/Oceanic	Taiwan	9 490
	Indonesia	603
	Thailand	495
	India	423
	Australia	282
	Viet Nam	223
	Malaysia	112
	Singapore	44
	Philippines	36
	Cambodia	29
Asia/Oceania	Taiwan	15 339
	Malaysia	186
	Indonesia	161
	India	150
	Japan	97
	Thailand	82
	Singapore	44
	Lao People’s Democratic Republic	26
	Philippines	22
	Viet Nam	18

Top 10 countries in terms of occurrence records for each continent are shown for *Ae.aegypti* (a) and *Ae.Albopictus*(b) (Source: Kraemer et al, eLife 2015;4:e08347, 10.7554/eLife.08347)

Currently, dengue cases are being reported in Tanzania with confirmed clinical cases and dengue haemorrhagic fever in patients who attended the Bombo dispensary (Bombo, Tanga), Hai hospital (Hai, Kilimanjaro), Tanganyika plantation company (TPC) hospital (Lower Moshi, Kilimanjaro) and Kilosa district hospital (Kilosa, Morogoro) [6, 7]. *Aedes aegypti* mosquitoes have been found to occupy habitats in both urban and rural environments [8], in highland and lowland areas [9]. In Tanzania, *Aedes* mosquitoes have often been found in lowlands [8] and have also recently been found to occupy the highlands (areas defined as having an altitude of 900 m above sea level), but with no dengue viruses [9] as they have in the lowlands of the country [8, 10].

The first cases of dengue were reported between 1823 and 1870 in Zanzibar archipelago, followed by outbreaks in mainland Tanzania between 2010 and 2014 [8]. Dar es Salaam, the main commercial and administrative hub, is prone to dengue outbreaks [8]; other cases have been reported in Tanga, Morogoro and Kilimanjaro [6, 7]. The previous outbreaks in Dar es Salaam were associated with circulating dengue virus serotype 2 (DENV2) [8]. An entomological study conducted by resident researchers reported a high density of *A. aegypti* mosquitoes spreading widely throughout Dar es Salaam and in the outskirts of the city [11].

The World Health Organization (WHO) has approved the first-ever dengue vaccine recently, however, it has not been widely deployed in control programmes in many countries in Sub-Saharan Africa [12]. The current findings show that CYD-TDV is safe and efficacious for 2–14 year-olds when given as part of a three-time dose programme [13]. The vaccine efficacy for the prevention of virologically confirmed dengue cases was found to be greater than the primary endpoint threshold, which is needed for the efficacious accepted point of the vaccine. The level of efficacy for all 25 months of follow-up for study participants who had received the vaccine in Latin America had, as expected, the highest performance outcome and was hence approved by the WHO [13]. Due to the higher efficacy among vaccinated patients aged nine years and above, a licence has been obtained in several countries, which have accepted the vaccine, for patients aged 9–60 years, the group that responded the best to the vaccine [14, 15]. There is evidence that the introduction of the CYD-TDV vaccine among adolescents in high dengue transmission zones on a routine basis will reduce the hospitalisation rate by about 10–30% [15]. Thus, *A. aegypti* vector control using insecticides remains the cornerstone of preventing dengue outbreaks.

Emergence and spread of vector insecticide resistance to the main classes of insecticides used in *A. aegypti* control is a serious operational impediment that could compromise the control of dengue and other vector-borne diseases. Although many studies have investigated the characterisation of malaria vector resistance, there is limited evidence on the *A. aegypti* insecticide resistance status in the region. Two mechanisms for insecticides resistance regarding *A. aegypti* mosquitoes are proposed: 1) increased activity of detoxification enzymes and glutathione S-transferases (GSTs); and (2) structural modifications in insecticide binding sites leading to a decreased affinity for the insecticide [16]. In addition, there is a suggestion of possible cross-resistance between organophosphate and pyrethroid insecticides mediated by polymorphisms in the voltage-gated sodium channel (knockdown resistance mutation) [16].

A few studies have been conducted on the effect of insecticides on natural mosquito populations in Sub-Saharan Africa, with one reporting reduced susceptibility to deltamethrin, lambda-cyhalothrin and propoxur in Western Africa (Dakar, Senegal) [17]. In Yaoundé, Cameroon, a study demonstrated that *A. albopictus* mosquitoes are probably resistant to deltamethrin, however, most of the *A. aegypti* population was shown to be susceptible to deltamethrin, propoxur and fenitrothion [18]. Little is known about the susceptibility status of the *A. aegypti* vector population in Eastern Africa. Elsewhere, resistance to pyrethroid-based insecticides has been shown in *A. aegypti* mosquitoes in several reports emanating from Asia, South America and Latin America [19–22]. Thus, for conventional insecticides to remain effective for dengue vector control, it is essential that the susceptibility status of *A. aegypti* vector populations is regularly monitored.

Furthermore, dengue infection transmission relies on the productivity of female *Aedes* mosquitoes from breeding habitats. Habitat productivity ultimately determines the number of adult vectors. The adult female *Aedes* mosquitoes emerging from breeding habitats are epidemiologically important because only female adults can feed on human hosts in contrast to the mosquito larvae and pupae density [23]. Several factors can influence productivity and abundance such as female oviposition preference, habitat type and size, and vegetation cover [23, 24]. Therefore, an estimation of the number of emerging *Aedes* adult mosquitoes is important to determine the productivity of mosquito breeding in their ecological habitats. Due to limited resources, the determination of the habitats that are most productive for targeted larval and adult control measures is of priority in Africa.

We adopted the WHO standard bioassay approach to investigate the resistance status of pyrethroid-based insecticides. The study aimed at investigating the phenotypic susceptibility status and habitat productivity of *A. aegypti* populations collected in different settings in Dar es Salaam, a city prone to seasonal dengue outbreaks.

## Methods

### Study site

**Fig. 1:**
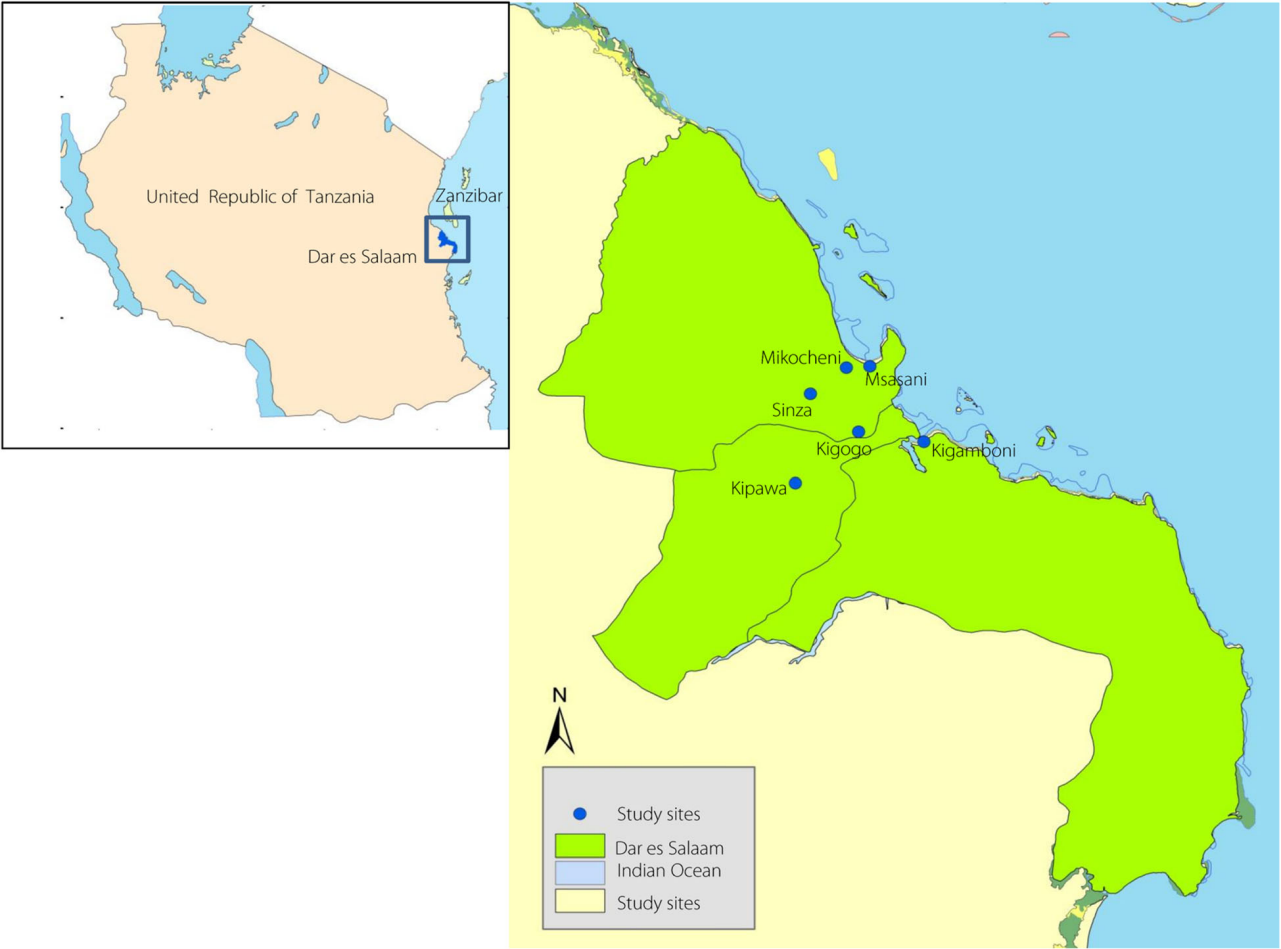
A map of Dar es Salaam, Tanzania showing the sampling sites of *Aedes aegypti* mosquitoes

The present study was conducted in six randomly selected wards in Dar es Salaam: Msasani, Mikocheni, Sinza, Kigogo, Kipawa and Kigamboni (Fig. 1). Dar es Salaam is one of the fastest growing cities in Sub-Saharan Africa, with a population growth of approximately 8% per year. It is the main administrative region and economic hub of Tanzania. The national census survey of 2012 indicates the city has a population of 4 364 541 people [25].

Dar es Salaam is located in the eastern part of the country at 6°52′S, 39°12′E, at 55 m above sea level. The average temperature is 25.9 °C, with the lowest and highest temperatures occurring in July – August and February – March, respectively [26]. The area is characterised by two rainy seasons: short rains (October to December) and long rains (March to May), with a total annual average precipitation of 1 148 mm. Relative humidity is high, reaching 100% almost every night throughout the year, but falling to 60% during the day. The city is characterised by unplanned, poor sanitation and a shortage of water, which leads to storing water in vessels or containers that are potential breeding habitats for *A. aegypti* mosquitoes.

### Habitat productivity and abundance

Mosquito larvae and pupae were collected from various containers and water storage vessels during the survey. The breeding habitats were recorded, including their locations and types. In relation to habitat productivity, each of the positive larval habitats, the pupae and larvae, were collected and placed in sample containers and transported to the laboratory for analysis and data recording. The pupae collected were placed in a paper cup kept in the insectary in order for the adult mosquito to emerge. The sex and species of the adults were identified based on the standard methods used by Banerjee et al. [27]. The number of females emerged was recorded for each habitat type and site sampled. Male mosquitoes were excluded because they are not a disease vector. The surface area of each habitat type sampled was estimated in square metres. Habitat productivity was determined by calculating the number of females emerged per square metre according to previous ecological studies conducted in western Kenya highlands [28].

### Sample collection and mosquito rearing

The sampling was done once a week from January to July 2015 in each selected site. The *A. aegypti* aquatic stages were collected using a dipper and pipette, and the geographical coordinates of each sampling site were recorded. The collected larvae and pupae were put in a container and transported immediately to the insectary where they were transferred into larvae rearing trays. The rearing was done under the standard conditions: temperature of 27 °C ± 1 °C and a relative humidity of 80% ± 10%, with the larvae being fed cat food pellets. The pupae collected from rearing trays were kept in mosquito cages sized 30 cm × 30 cm × 30 cm until emergence. A 10% sucrose solution was prepared and used for feeding the emerged adults before the susceptibility test; this took 3–5 days depending on the number of adults needed. The emerged adult mosquitoes were sorted by separating the males from the females, and then the females were subjected to insecticide susceptibility testing.

### Adult bioassays for insecticide susceptibility tests

The susceptibility test was performed according to the WHO guidelines using the emerged *A. aegypti* females, aged three days, from different sites. Batches of 20 non-blood fed female *A. aegypti* mosquitoes were aspirated in WHO holding tubes lined with untreated paper for one hour. They were then exposed to insecticides and the outcomes were recorded for each mosquito larvae sampled. Thereafter, they were provided with 10% of sucrose solution for 24 h after been exposed to insectcides before scoring mortality. Mosquitoes were transferred from the holding tubes to the WHO exposure tubes, which were lined with paper impregnated with the relevant pyrethroid insecticides (treatments). For each exposure, four treated tubes of the same insecticide and two control replicate tubes were used. The insecticides were deltamethrin (0.05%), permethrin (0.75%) and lambda-cyhalothrin (0.05%). In the exposure kits, mosquitoes were exposed for 60 min and the number of mosquitoes knocked down was recorded at the following time points: 10, 15, 20, 30, 40, 50 and 60 min. Mosquitoes were then transferred to paper cups and provided with 10% sucrose solution for recovery monitoring at 26.0 °C ± 1.0 °C and 80% ± 10% humidity for 24 h. For each insecticide, there were five replicates: four treatment replicates and one control. The mortality was concluded 24 h post insecticide exposure.

### Data analysis

Data were entered into Microsoft Excel (American Multinational Technology Company, Redmond, Washington) and transferred to SPSS version 18.0 (SPSS Inc., Chicago, IL). Larval and pupal abundances were computed using habitat type and sampling sites as factors with analysis of variance one-way analysis (ANOVA). Data were log transformed before analysis, as the data had great variations between habitats. To assess the susceptibility status, bioassays for different insecticides per site were done as according to WHO recommendations [29]: if 98–100% mosquito mortality is observed, this indicates insecticide susceptibility, mortality < 98% suggests existence of resistance that needs to be confirmed, and mortality < 90% suggests resistance [29]. Controls were also set up by systematically exposing a group of mosquitoes to untreated papers. The test results were discarded if mortality in the control group was over 20% but corrected if mortality was between 5 and 20% using Abbot’s formula [30]. To calculate the bioassays percentage knockdown times and mortality at 50 and 95% of the population (KDT_50_ and KDT_95_), probit analysis incorporating regression models were applied. The habitat productivity was calculated by counting the number of adult females (disease transmitters) emerged in each habitat and divided by the surface area of that habitat by sampling site. As appropriate, means, standard errors, 95% confidence intervals (*CI*s) and variances for all variables were calculated. A statistical significance level was set at *P*-value ≤ 0.05.

## Results

**Fig. 2:**
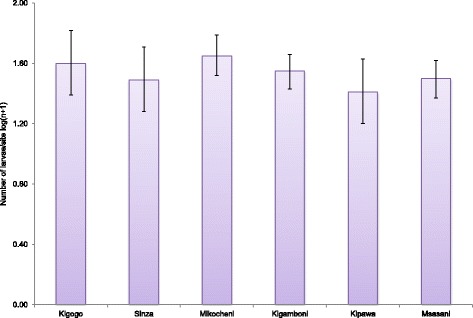
Larval density as observed at the different sites in Dar es Salaam, Tanzania

A total of 17 461 immature mosquitoes (larvae and pupae) were obtained from the six study sites in Dar es Salaam from January to July 2015. During the collection period, discarded tins, tyres and water storage vessels were found to harbour a high abundance of *A. aegypti* larvae at the sites. The distribution of the *A. aegypti* larvae breeding sites are presented in Fig. 2. Larval abundance among the three habitat types (discarded tins, discarded car tyres and water storage vessels) was not statistically different (degree of freedom (*df*) = 2, *F*-test (*F*) = 1.174, *P* = 0.311). Similarly, pupal abundance was not statistically different (*df* = 2, *F* = 0.919, *P* = 0.400). When compared between the study sites, the larvae were equally high in all sites (*df* = 5, *F* = 1.036, *P* = 0.397) with a similar trend observed for pupal abundance (*df* = 5, *F* = 1.952, *P* = 0.086).

### *Aedes aegypti* habitat productivity

**Fig. 3:**
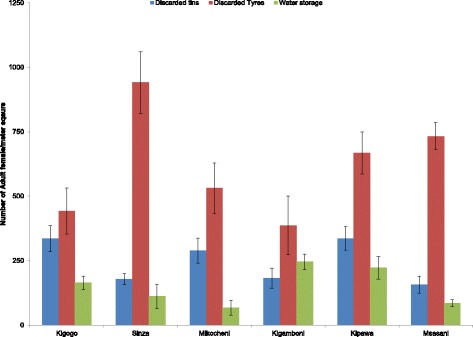
Habitat productivity of *Aedes aegypti* mosquitoes at the three breeding habitats in the six wards in Dar es Salaam, Tanzania

**Fig. 4:**
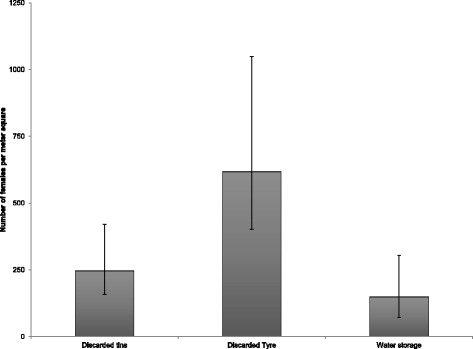
Female *Aedes aegypti* habitat productivity at the different breeding habitats in Dar es Salaam, Tanzania

Figures 3 and 4 illustrate the productivity of *A. aegypti* mosquitoes at the different study sites. Adult productivity was highest in disposed tyres (*P* < 0.002), while water storage tanks had the lowest productivity (*P* < 0.0004). Our findings suggest a consistent pattern of productivity across the sites (see Fig. 3).

### Susceptibility status of adult *A. aegypti* mosquitoes to insecticides

#### Adult bioassays

The susceptibility status of populations of *A. aegypti* mosquitoes to different concentrations of pyrethroid insecticides is shown in Table 2.

**Table 2:** Knock-down times and mortality rates of field collected *Aedes aegypti* mosquitoes exposed to 0.05% lambdacyhalothrin, 0.75% permethrin and 0.05% lambdacyhalothrin using WHO standard bioassay

Insecticide	Site	(*N*)	Replicates	Mean mortality (%) ± *SD*	KDT50 (Min.)	95% *CI*	KDT95 (Min.)	95% *CI*	Status
0.05% Deltamethrin	Kigogo	160	8	90.6 ± 1.8	30	24.6–36.8	90.9	64.6–178.9	R*
	Kipawa	160	8	92.5 ± 0.9	24.9	21.7–28.3	55.2	45.8–73.7	R*
	Msasani	160	8	87.5 ± 1.3	29.9	24.0–30.1	78.7	64.1–105.9	R
	Sinza	160	8	96.8 ± 0.9	26	22.8–29.4	60.1	49.8–80.1	R*
	Mikocheni	160	8	86.3 ± 1.9	24.9	21.3–28.6	58.5	47.4–82.3	R
	Kigamboni	160	8	91.9 ± 1.1	30.3	27.0–33.9	65.9	55.1–86.4	R*
0.75% Permethrin	Kigogo	160	8	86.8 ± 1.2	30.9	28.1–34.0	68.1	58.2–85.4	R
	Kipawa	160	8	83.1 ± 2.1	34.4	30.6–39.0	94.6	74.9–35.9	R
	Msasani	160	8	85.0 ± 1.3	32.1	27.9–36.9	71.2	57.4–102.5	R
	Sinza	160	8	96.2 ± 0.9	24.3	21.0–27.6	54.3	44.8–73.4	R*
	Mikocheni	160	8	88.1 ± 1.4	33.7	28.1–41.1	89.3	65.3–164.2	R
	Kigamboni	160	8	91.2 ± 1.0	29.3	27.0–31.7	67.1	58.6–80.4	R*
0.05% Lambdacyhalothrin	Kigogo	160	8	86.3 ± 1.4	29.2	27.9–30.6	67.2	61.8–74.1	R
	Kipawa	160	8	85.6 ± 2.1	26.7	22.7–31.0	64.5	51.5–94.1	R
	Msasani	160	8	85.0 ± 0.7	32.8	31.5–34.3	69	63.9–75.7	R
	Sinza	160	8	83.8 ± 0.7	30.7	27.5–34.3	76	62.8–101.1	R
	Mikocheni	160	8	84.4 ± 0.6	27.9	25.6–30.4	69.2	59.8–84.2	R
	Kigamboni	160	8	83.1 ± 0.7	29.3	27.9–30.7	69.6	64.8–77.2	R

*N* number of samples, *CI* confidence interval, *SD* standard deviation, *KDT* knock-down time, *KDT50* time taken for 50% of the test mosquitoes to knock down, *KDT95* time taken for 95% of the test mosquitoes to knock down. S; Full susceptible (observed mortality 98–100%) R*; Suspected resistance needs to be confirmed (mortality 90–97%) and R; Resistance (observed mortality < 90%)

Resistance to 0.05% deltamethrin was detected in the Msasani and Mikocheni collected strains and the mean mortality rates 24 h post exposure were 87.5 and 86.3%, respectively. The samples collected from the remaining sites were found to have suspected resistance to 0.05% deltamethrin that needs further investigation.

The results of 0.75% permethrin bioassays suggest resistance in almost all sites with the exception of two sites (Sinza and Kigamboni), where resistance is suspected with a mortality rate of above 90% but less than 98%. The mean mortality rates 24 h post exposure for 0.75% permethrin ranged between 83.1 and 96.2% (see Table 2). The mortality rates pertaining to *Aedes* mosquitoes collected from Kigamboni (91.2%) and Sinza (96.2) were relatively high and classified as suspected resistance.

For *A. aegypti* mosquitoes subjected to 0.05% lambda-cyhalothrin, the mortality recorded ranged from 83.1 to 86.3%. Full susceptibility to 0.05% lambda-cyhalothrin was recorded in mosquitoes from all six sites (see Table 2).

#### Knockdown times

For 0.05% deltamethrin bioassays, the KDT_50_ ranged from 24.9 to 30.3 min, while KDT_95_ ranged from 55.2 to 90.9 min. The highest KDT_50_ (30.3 min) was observed in mosquitoes collected in Kigamboni (see Table 2).

The KDT_50_ values were comparable across the sites, ranging from 24.3 to 34.4 min, while the KDT_95_ ranged from 54.3 to 94.6 min for 0.75% permethrin. The longest KDT_50_ (34.4 min) was recorded in Kipawa [95% *CI*: 30.6–39.0] (see Table 2).

For 0.05% lambda-cyhalothrin, the observed KDT_50_ and KDT_95_ ranged between 26.7 to 32.8 min and 64.5 to 69.2 min, respectively. Overall, it took a long time for mosquitoes to be knocked down (32.8 min) by lambda-cyhalothrin in Msasani compared with the other sites (see Table 2).

## Discussion

Despite the development of a recent dengue vaccine (Dengvaxia®) and its endorsement by the WHO, the vaccine is still unavailable in Sub-Saharan countries. Therefore, vector control remains the cornerstone of dengue prevention and control. Other insecticides are an integral part of vector control, however, the paucity of data on the susceptibility status of *A. aegypti* mosquitoes could compromise the effectiveness of dengue vector control campaigns. In this study, we examine data on *A. aegypti* productivity and the mosquito population’s pyrethroid susceptibility status in Dar es Salaam, a city experiencing frequent outbreaks of dengue fever.

The breeding habitats included in this study were those which were positively infested with *A. aegypti* larvae. The habitats found to have *Aedes* larvae were disposed tyres, water storage containers and discarded tins. Tyres and water storage containers located outdoors appeared to be the most stable breeding habitats for *A. aegypti* mosquitoes. Discarded tyres are used for various purposes including fencing and in car garages, and they are found in many places. In addition, plastic water storage vessels of various sizes also contribute to the abundance of breeding habitats. Most households keep water storage vessels due to water scarcity and irregular water shortages. Our findings are consistent with a previous study that also reported that discarded tyres have a high abundance of *A. aegypti* larvae in Dar es Salaam [11]. Similarly, a recent study found that most of the water storage vessels in most of the households in the same setting harboured larvae or pupae of *Aedes* spp. [8].

Regarding *A. aegypti* female adult productivity, our data suggest a similar pattern of habitat productivity across all sites. However, tyres had the highest productivity, while water storage containers had the lowest productivity among the observed breeding habitats. This also compares with other findings that documented high productivity in discarded car tyres [8, 11]. However, contrary to our finding, a study conducted in the Philippines and Malaysia on habitat productivity demonstrated that domestic containers were the most productive and targeted for *A. aegypti* control, which have added value to dengue control in these countries [6, 7]. Other breeding habitats have also been documented, including self-watering pots and domestic waste disposal containers, which was not observed in the present study [11]. Nonetheless, our data suggest similar female *A. aegypti* productivity in habitats as also reported in other settings [24, 27, 31]. This demonstrates that for effective reduction and elimination of the most productive *A. aegypti* mosquitoes, campaigns should also target water storage containers to reduce mosquito density. Our study was, however, limited by the fact that seasonal productivity and influence of other climatic variables could not be explored, and this is worth exploring in future studies.

The susceptibility test results of dengue vectors generally demonstrated that *A. aegypti* populations from Dar es Salaam subjected to lambda-cyhalothrin had the highest level of resistance in all six study sites, with a mortality rate of less than 86%, which shows increased resistance. However, the WHO recommends further investigation on the mechanisms and distribution of resistance to be undertaken if the observed mortality is between 90 and 96% [29]. Of the three insecticides tested for susceptibility, deltamethrin showed the highest mortality rate, while permethrin showed a moderate mortality rate and lambda-cyhalothrin showed the lowest mortality rate. Similar results regarding resistance to pyrethroids were found by Marcombe et al. [20, 21]. The level of susceptibility varied according to the insecticide used and sites. *A. aegypti* resistance to lambda-cyhalothrin seems to have increased across the sites compared with the other insecticides examined in this study. The reason as to why lambda-cyhalothrin had developed higher levels of Insecticide resistance compared to the others is merely associated with the intensive use of lambda-cyhalothrin in conventionally treated bed nets in Tanzania [32]. However, possible cross-resistance with insecticides used in malaria control is also speculated. To our knowledge, this is the first study to demonstrate the susceptibility status of *A. aegypti* against different registered pyrethroids in Tanzania and there are limited data to compare our findings with. In other countries, studies have found detailed mechanisms that are involved in the different insecticides resistance, which is also needed to be done in Tanzania for strategic control of *A. aegypti* as in Thailand and Brazil [33, 34].

Mosquito populations from Mikocheni, Kipawa, Kigogo and Kigamboni showed high resistance to all three insecticides. Mikocheni had the lowest resistance, but mosquitoes from Sinza showed a susceptibility to deltamethrin and permethrin, with mortalities of 97 and 96% respectively, and indicators of resistance to lambda-cyhalothrin. The high resistance among *Aedes* mosquito populations can be attributed to the prolonged use of these insecticides in controlling mosquitoes domestically, such as with sprays, coils and long-lasting insecticidal nets (LLINs). Pyrethroids have also been widely used in agriculture [35–38]. The frequent use of insecticides for vector control has led to increasing concerns over the development of insecticide resistance of these vectors on the environment and human health, which can compromise vector control strategies. The rapid spread of the *Aedes* vector due to transportability of either adult *Aedes* through vehicles or dried but viable eggs through containers could influence the spread and outbreak of dengue infections in non-endemic areas of Tanzania.

## Conclusions

This study has for first time shown an evidence-based spread of pyrethroid-based insecticide resistance in *A. aegypti* populations in Dar es salaam, Tanzania. This calls for a countrywide survey to be conducted to assess the susceptibility status of *A. aegypti* for better management of vector and all arboviruses transmitted by this vector.

The findings suggest that the high habitat productivity recorded in discarded tyres needs to be targeted for outbreak prevention and for controlling dengue fever infections. Our data suggest that *A. aegypti* populations in most of the sites in Dar es Salaam are fully resistant to permethrin and lambda-cyhalothrin, while deltamethrin demonstrated suspected resistance. The documented differences in the resistance profiles of *A. aegypti* mosquitoes warrant regular monitoring to elucidate the pattern concerning resistance against pyrethroid-based insecticides and define dengue vector control strategies.

## Additional files

Additional file 1: Multilingual abstracts in the five official working languages of the United Nations. (PDF 894 kb)

Additional file 2: The habitat larval and pupae abundance, physical chemical properties of habitat in different wards of Dar-es-salaam , Tanzania. (XLS 94 kb)

## Abbreviations

*CI*: Confidence Interval
COSTECH: Commission for Science and Technology
*df*: Degree of Freedom
*F*: *F*-test
KDT_50_: Knockdown time for 50% of the population
KDT_95_: Knockdown time for 95% of the population
NRF: National Research Foundation
WHO: World Health Organisation
