# Supplementary material for: Habitat productivity and pyrethroid susceptibility status of Aedes aegypti mosquitoes in Dar es Salaam, Tanzania
Source: Infect Dis Poverty. 2017 Jun 9;6:102. doi: 10.1186/s40249-017-0316-0 (PMC5465599; doi:10.1186/s40249-017-0316-0)
Supplement: Supplementary file 1 — Multilingual abstracts in the five official working languages of the United Nations. (PDF 894 kb) [file 40249_2017_316_MOESM1_ESM.pdf]

Translation of the abstract into the five official working languages of the United Nations

إنتاجية الموائل ووضع قابلية بيريثرويد من الزاعجة المصرية البعوض في دار السلام، تنزانيا

ليا ماتياس فيتو باراكا، أنيتا فيلبرت، استر إنوسينت، فيلبرت فرانسيس، جامبا نكوينجوليلا، إيلينيجايا ج. كويكا

ملخص

خلفية: الزاعجة المصرية (ذوات الجناحين: البعوضيات) هي الناقل الأساسي لفيروس حمى الضنك على المستوى العالمي. وتستند مكافحة نواقل حمى الضنك بشكل رئيسي على تخفيض عدد البعوض الناقل للداء من خلال التدخلات التي تستهدف الأماكن المحتملة لتكاثرها. ومع ذلك، في تنزانيا، لا يعرف إلا القليل عن حالة تكاثر البعوض الناقل للداء وقابلية الحشرات لدعم التنفيذ القائم على الأدلة لتدابير الرقابة. هدفت الدراسة إلى تقييم حالة الإنتاجية وقابلية بعوض الزاعجة المصرية للمبيدات الحشرية ذات القاعدة البيريثرويدية في دار السلام بتنزانيا.

أساليب: أجري التقييم الحشري بين يناير ويوليو 2015 في ستة أقسام تم اختيارها عشوائيا في دار السلام بتنزانيا. تم تحديد إنتاجية الموئل من جانب عدد من الإناث البالغات لبعوض الزاعجة المصرية الذي ظهر في المتر المربع الواحد. وضع قابلية للإناث البالغة من بعوضالزاعجة المصريةبعد التعرض إلى 0.05٪ الدلتاميثرين، 0.75٪ البيرميثرين و 0.05٪ لامدا سيهالوثرين تم التقييم باستخدام معيار بروتوكولات منظمة الصحة العالمية. سجلت معدلات وفيات بعد 24 ساعة من التعرض وكانت قد سجلت تأثير ضربة قاضية في نقطة زمنية من 10، 15، 20، 30، 40، 50 و 60 دقيقة لحساب أوقات الضربة القاضية المتوسطة (KDT$_{50}$ وKDT$_{95}$).

النتائج: تشير النتائج إلى أن الإطارات التي يتم التخلص منها كانت أعلى إنتاجية، بينما كان خزانات المياه أقل إنتاجية بين موائل تكاثر بعوض الزاعجة المصرية. أظهرت جميع المواقع انخفاض القابلية للدلتاميثرين (0.05٪) خلال 24 ساعة بعد التعرض، مع فيات تتراوح بين 86.3 ± 1.9 (متوسط ± SD) إلى 96.8 ± 0.9 (متوسط ± SD). وسجلت أدنى وأعلى حساسيات في قطاعي ميكوتشيني وسينزا، على التوالي. وبالمثل، أظهرت جميع المواقع انخفاض قابلية البيرميثرين (0.75٪) تتراوح ما بين 83.1 ± 2.1٪ (متوسط ± SD) إلى 96.2 ± 0.9٪ (متوسط ± SD)، في كيباوا وسينزا، على التوالي. نسبيا لوحظت معدلات وفيات منخفضة فيما يتعلق بلامدا سيهالوثرين (0.05٪) في جميع المواقع، التي تتراوح بين 83.1 ± 0.7 (متوسط ± SD) إلى 86.3 ± 1.4 (متوسط ± SD). ومتوسط KDT$_{50}$ للدلتاميثرين، البيرميثرين ولامدا سيهالوثرين كان 24،9-30،3 دقائق، 24،3-34،4 دقائق و26،7-32،8 دقيقة، على التوالي. وKDT$_{95}$ كانت 55،2-90،9 دقائق لالدلتاميثرين، 54،3-94،6 دقائق للبيرميثرين و64،5-69،2 دقائق للامدا سيهالوثرين.

الاستنتاجات: <bx/>الموائل المنتجة لبعوض الزاعجة المصرية (أ) في دار السلام كانت صهاريج تخزين المياه، والمعلبات المهملة، وإطارات السيارات. انخضت قابلية بعوض الزاعجة المصرية في مقاومة المبيدات الحشرية ذات القاعدة البيريثرويدية. الاختلافات الموثقة في شكل المقاومة لبعوض الزاعجة المصرية يحتاج إجراء مراقبة عادية لنمط المقاومة ضد المبيدات الحشرية ذات القاعدة البيريثرويدية وتحديد استراتيجيات مكافحة نواقل حمى الضنك.

Translated from English version into Arabic by Mahmoud Sami, through

TRANSLATORS WITHOUT BORDERS

坦桑尼亚达累斯萨拉姆地区埃及伊蚊的栖息地繁殖能力和拟除虫菊酯敏感性状况

Leah Mathias, Vito Baraka, Anitha Philbert, Ester Innocent, Filbert Francis, Gamba Nkwengulila, Eliningaya J. Kweka

摘要

引言：埃及伊蚊（*Aedes aegypti*）是全球登革热病毒的主要媒介。登革热的媒介控制主要通过以潜在的繁殖地点为目标的干预措施来减少媒介群体数量。然而，在坦桑尼亚，很少有人知道这种媒介在栖息地繁殖能力和对杀虫剂易感性状态，进而支持以证据为基础的防制措施。本研究旨在评估坦桑尼亚达累斯萨拉姆地区埃及伊蚊的栖息地繁殖能力和拟除虫菊酯敏感性状况。

方法：于2015年1月至7月在坦桑尼亚达累斯萨拉姆随机选择的6个点进行昆虫学评估。栖息地繁殖能力由每平方米出现的雌性成年埃及伊蚊数量计算。使用WHO标准方案评估雌性成蚊在暴露于0.05％溴氰菊酯、0.75％氯菊酯和0.05％-λ氯氟氰菊酯后的敏感性状态。在暴露h 24后记录蚊虫死亡率，计算接触10、15、20、30、40、50和min 60时的击倒情况，计算半数击倒时间（KDT_{50}和KDT_{95}）。

结果：结果表明，埃及伊蚊在废弃轮胎中繁殖能力最高，而在储水罐中繁殖能力最低。所有调查点在暴露后h 24内表现出对溴氰菊酯的敏感性降低（0.05％），死亡率为%1.9±%86.3至%0.9±%96.8。敏感性最低和最高的分别在Mikocheni和Sinza。类似地，在Kipawa和Sinza，所有调查点也出现了对氯菊酯敏感性的降低（0.75％），死亡率为为2.1±%83.1％至0.9±%96.2％。相对而言，所有调查点蚊虫对-λ三氯氟氰菊酯（0.05％）的死亡率较低，范围为%0.7±%83.1至%1.4±%86.3。溴氰菊酯、氯菊酯和-λ氯氟氰菊酯的KDT_{50}中位数分别为min 30.3-24.9，-24.3 min 34.4和min 32.8-26.7。溴氰菊酯的KDT_{95}为min 90.9-55.2，氯菊酯的为min 94.6-54.3，-λ氯氟氰菊酯的为min 69.2-64.5。

结论：在达累斯萨拉姆发现的埃及伊蚊的繁殖性栖息地为储水容器，废弃的罐和轮胎。埃及伊蚊对拟除虫菊酯敏感性有所降低并出现抗性。对于观察到的埃及伊蚊抗性资料的差异需要进一步对拟除虫菊酯的抗性进行定期监测并制定相应的登革热媒介控制策略。

Translated from English version into Chinese by Xin-Yu Feng, edited by Pin Yang

CHINESE CENTER FOR DISEASE CONTROL AND PREVENTION
NATIONAL INSTITUTE OF PARASITIC DISEASES

La productivité de l'habitat et l'état de la sensibilité aux pyréthrinoïdes chez les moustiques *Aedes aegypti* à Dar es Salaam, Tanzanie

Leah Mathias, Vito Baraka, Anitha Philbert, Ester Innocent, Filbert Francis, Gamba Nkwengulila and Eliningaya J. Kweka

RÉSUMÉ

Contexte : *Aedes aegypti* (Diptera: Culicidae) est le principal vecteur du virus de la dengue dans le monde. Le contrôle du vecteur de la Dengue est basé principalement sur la réduction de la population de vecteurs grâce à des interventions qui ciblent les sites de reproduction potentiels. Cependant, en Tanzanie, on sait peu sur la productivité de l'habitat et l'état de la sensibilité aux insecticides de ce vecteur pour soutenir la mise en œuvre fondée sur les preuves de mesures de contrôle. La présente étude visait à évaluer l'état de la productivité et de la sensibilité des moustiques *A. aegypti* aux insecticides à base de pyréthrinoïdes à Dar es Salaam, en Tanzanie.

Méthodes: Une évaluation entomologique a été menée entre janvier et juillet 2015 dans six quartiers, choisis au hasard, à Dar es Salaam, en Tanzanie. La productivité de l'habitat a été

déterminée par le nombre de femelles adultes de *A. aegypti* ont émergé par mètre carré. L'état de la sensibilité des femelles adultes de *A. aegypti* après une exposition à 0,05 % de deltaméthrine, 0,75 % de perméthrine et 0,05 % de lambda-cyhalothrine a été évalué en utilisant les protocoles normalisés de l'OMS. Les taux de mortalité ont été enregistrés après 24 heures d'exposition et l'effet choc ont été enregistrés à intervalles de temps de 10, 15, 20, 30, 40, 50 et 60 minutes afin de calculer la médiane des intervalles du choc (KDT_{50} et KDT_{95}).
Résultats: Les résultats suggèrent que parmi les habitats de reproduction des moustiques du genre de *A. aegypti*, les pneus jetés favorisent la productivité la plus élevée, tandis que les réservoirs de stockage d'eau favorisent la productivité. Tous les sites ont démontré une sensibilité réduite à la deltaméthrine (0,05 %) pendant 24 heures après l'exposition, avec des mortalités allant de 86,3 ± 1,9 (moyenne ± écart-type) à 96,8 ± 0,9 (moyenne ± écart-type). Les sensibilités les plus bases et les plus élevées ont été enregistrées dans les quartiers de Mikocheni et de Sinza, respectivement. De même, tous les sites ont démontré une réduction de la sensibilité à la perméthrine (0,75 %) allant de 83,1 ± 2,1 % (moyenne ± écart type) à 96,2 ± 0,9 % (moyenne ± écart-type), à Kipawa et Sinza, respectivement. Des taux de mortalité relativement faibles ont été observés par rapport à la lambda-cyhalothrine (0,05 %) dans tous les sites, allant de 83,1 ± 0,7 (moyenne ± écart-type) à 86,3 ± 1,4 (moyenne ± écart-type). Les médianes KDT_{50} pour la deltaméthrine, la perméthrine et la lambda-cyhalothrine étaient de 24,9 - 30,3 minutes, 24,3 - 34,4 minutes et 26,7 - 32,8 minutes, respectivement. Les médianes de KDT_{95} étaient de 55,2 - 90,9 minutes pour la deltaméthrine, 54,3 - 94,6 minutes pour la perméthrine et 64,5 - 69,2 minutes pour la lambda-cyhalothrine.
Conclusions : Les source d'habitats reproductifs favorables aux moustiques du genre de *A. aegypti* trouvés à Dar es Salaam étaient des conteneurs de stockage d'eau, des boîtes et des pneus jetés. Il y avait une sensibilité réduite chez les *A. aegypti* à et une émergence de la résistance aux insecticides à base de pyréthrinoïdes. Les différences documentées dans les profils de résistance chez les moustiques du genre de *A. aegypti* garantissent une surveillance régulière du type de résistance aux insecticides à base de pyréthrinoïdes et définissent des stratégies de lutte contre les vecteurs de la dengue.

Translated from English version into French by Ejila Makangu, through

TRANSLATORS
WITHOUT BORDERS

Плодовитость в естественной среде обитания и восприимчивость к воздействию пиретроида комаров вида *Aedes aegypti* в Дар-эс-Саламе, Танзания

Лиа Матиас, Вито Барака, Анита Фильберт, *Эстер Иносент, Фильбер Фрэнсис, Гамба Нквенгулила, Элинингайа Дж. Квека*

АННОТАЦИЯ

Справка: *Aedes aegypti или комар жёлтолихорадочный* (Diptera: Culicidae) является основным переносчиком вируса денге во всем мире. Борьба с переносчиками

лихорадки денге основывается, преимущественно, на сокращении популяции переносчиков посредством вмешательства, нацеленного на потенциальные места размножения. Тем не менее, в Танзании собрано мало данных о плодовитости комаров данного вида в естественной среде обитания и их восприимчивости к инсектицидам для обоснования и осуществления мер контроля на основе фактических данных. Настоящее исследование направлено на оценку плодовитости комаров вида *A. Aegypti* и их восприимчивости к инсектицидам на основе пиретроидов в Дар-эс-Саламе, Танзания.

Методы: энтомологических оценка проводилась в период с января по июль 2015 года в шести произвольно выбранных округах Дар-эс-Салама, Танзания. Плодовитость определялась по количеству встреченных на квадратный метр взрослых самок вида *A. Aegypti* . Восприимчивость взрослых самок вида *A. Aegypti* к воздействию 0,05% дельтаметрину, 0,75% перметрину и 0,05% лямбда-цигалотрину оценивалась с использованием стандартных протоколов ВОЗ. Показатели смертности регистрировались через 24 часа после воздействия, нокдаун-эффект фиксировался на временных точках 10, 15, 20, 30, 40, 50 и 60 минут, чтобы вычислить медиану времени нокдаун (KDT_{50} и KDT_{95}).

Результаты: Полученные результаты свидетельствуют о том, что самая высокая плодовитость наблюдается у популяций комаров, живущих в использованных шинах, в то время как популяции, живущие в резервуарах для хранения воды имеют наиболее низкую плодовитость среди всех мест обитания вида *A. Aegypti.* Все исследуемые точки продемонстрировали пониженную восприимчивость к дельтаметрину (0,05%) в течение 24 часов после воздействия, с уровнем смертности в пределах от 86,3 ± 1,9 (среднее ± стандартное отклонение) до 96,8 ± 0,9 (среднее ± стандартное отклонение). Наиболее низкие и высокие показатели восприимчивости были зафиксированы в округах Микочени (Mikocheni) и Синза (Sinza), соответственно. Аналогичным образом, все исследуемые точки продемонстрировали пониженную восприимчивость к перметрину (0,75%) в диапазоне от (среднее ± стандартное отклонение) до 96,2 ± 0,9% (среднее ± стандартное %2,1 ± 83,1 отклонение) в округах Кипава (Kipawa) и Синза (Sinza), соответственно. Относительно низкие показатели смертности наблюдались при использовании лямбда-цигалотрина на всех исследуемых точках от 83,1 ± 0,7 (среднее ± стандартное отклонение) до 86,3 (%0,05) (среднее ± стандартное отклонение). Медианы KDT_{50} для дельтаметрина, перметрина 1,4 ± и лямбда-цигалотрина составляли 24,9 - 30,3 минуты, 24,3 - 34,4 минуты и 26,7 - 32,8 минуты, соответственно. Медианы KDT_{95} составляли 55,2 - 90,9 минут для дельтаметрина, .минуты для перметрина и 64,5 - 69,2 минут для лямбда-цигалотрина 94,6 - 54,3

Выводы: Наиболее благоприятными с точки зрения плодовитости места обитания вида *A. Aegypti*, обнаруженными в Дар-эс-Саламе, были резервуары для хранения воды, выброшенные банки и шины. Наблюдалось снижение восприимчивости и появление устойчивости у комаров вида *A. Aegypti* к инсектицидам на основе пиретроидов. Зафиксированные различия в профилях сопротивляемости *A. Aegypti* служат основанием для регулярного мониторинга устойчивости к инсектицидам на основе пиретроидов и .определяют направления борьбы с переносчиками лихорадки денге

Translated from English version into Russian by datran, through

Productividad del hábitat y estado de susceptibilidad a piretroides de los mosquitos *Aedes aegypti* en Dar es Salaam, Tanzania

Leah Mathias, Vito Baraka, Anitha Philbert, Ester Innocent, Filbert Francis, Gamba Nkwengulila, Eliningaya J. Kweka

RESUMEN

Antecedentes: *Aedes aegypti* (Diptera: Culicidae) es el principal vector del virus del dengue a nivel mundial. El control de los vectores del dengue se basa principalmente en la reducción de la población de vectores mediante intervenciones dirigidas contra los sitios potenciales de reproducción. Sin embargo, en Tanzania, poco se sabe sobre la productividad del hábitat de este vector y el estado de susceptibilidad a insecticidas como para fundamentar la implementación de medidas de control en base a la evidencia. El presente estudio tuvo como objetivo evaluar la productividad y el estado de susceptibilidad a insecticidas a base de piretroides de los mosquitos *A. aegypti* en Dar es Salaam, Tanzania.

Métodos: Se llevó a cabo una evaluación entomológica entre enero y julio del año 2015 en seis salas hospitalarias seleccionadas al azar en Dar es Salaam, Tanzania. Se determinó la productividad del hábitat por la cantidad de mosquitos *A. aegypti* hembras adultas que emergieron por metro cuadrado. Se evaluó el estado de susceptibilidad de las hembras adultas de *A. aegypti* luego de ser expuestas a deltametrina al 0,05%, permetrina al 0,75% y lambda cialotrina al 0,05% mediante el uso de protocolos estándar de la OMS. Se registraron las tasas de mortalidad luego de 24 horas de exposición y se registró el efecto derribo a los puntos temporales de 10, 15, 20, 30, 40, 50 y 60 minutos para calcular los tiempos promedio de derribo (KDT_{50} y KDT_{95}).

Resultados: Los resultados sugieren que los neumáticos desechados tienen la mayor productividad, mientras que los tanques de almacenamiento de agua tienen la productividad más baja entre los hábitats de reproducción de mosquitos *A. aegypti*. Todos los sitios demostraron susceptibilidad disminuida a la deltametrina (0,05%) dentro de las 24 horas pos exposición, con mortalidad que variaba entre 86,3 ± 1,9 (media ± desviación estándar) y 96,8 ± 0,9 (media ± desviación estándar). Las susceptibilidades más altas y más bajas se registraron en las salas de Mikocheni y Sinza, respectivamente. De manera similar, todos los sitios demostraron susceptibilidad disminuida a la permetrina (0,75%) variando entre 83,1 ± 2,1% (media± desviación estándar) y 96,2 ± 0,9% (media± desviación estándar), en Kipawa y Sinza, respectivamente. Se observaron tasas de mortalidad relativamente bajas con relación a la lambda cialotrina (0,05%) en todos los sitios, variando entre 83,1 ± 0,7 (media ± desviación estándar) y 86,3 ± 1,4 (media ± desviación estándar). Los KDT_{50} medios para la deltametrina, permetrina y lambda cialotrina fueron de 24,9 – 30,3 minutos, 24,3 – 34,4 minutos y 26,7 – 32,8 minutos, respectivamente. Los KDT_{95} fueron de 55,2 – 90,9 minutos para la deltametrina, 54,3 – 94,6 minutos para la permetrina y 64,5 – 69,2 minutos para la lambda cialotrina.

Conclusiones: Se encontró que los hábitats productivos para mosquitos *A. aegypti* en Dar es Salaam fueron los contenedores para almacenamiento de agua y las latas y neumáticos desechados. Hubo susceptibilidad disminuida de *A. aegypti* y emergencia de resistencia a los insecticidas a base de piretroides. Las diferencias documentadas en los perfiles de resistencia de los mosquitos *A. aegypti* justifican el monitoreo periódico de los patrones de resistencia a los insecticidas a base de piretroides y la definición de estrategias de control para el vector del dengue.

Translated from English version into Spanish by Maria Alejandra Aguada, through

TRANSLATORS
WITHOUT BORDERS
